# Seven facts and five initiatives for gut microbiome research

**DOI:** 10.1007/s13238-020-00697-8

**Published:** 2020-03-14

**Authors:** Danyi Li, Chunhui Gao, Faming Zhang, Ruifu Yang, Canhui Lan, Yonghui Ma, Jun Wang

**Affiliations:** 1 Beijing Rexinchang Biotechnology Research Institute Co. Ltd, Beijing, 100011 China; 2 Medical Center for Digestive Diseases, The Second Affiliated Hospital of Nanjing Medical University, Nanjing, 210011 China; 3 Key Lab of Holistic Integrative Enterology, Nanjing Medical University, Nanjing, 210011 China; 4 Division of Microbiotherapy, Sir Run Run Shaw Hospital, Nanjing Medical University, Nanjing, 211166 China; 5 State Key Laboratory of Pathogen and Biosecurity, Beijing Institute of Microbiology and Epidemiology, Beijing, 100071 China; 6 Centre for Bioethics, Medical College, Xiamen University, Xiamen, 361102 China; 7 CAS Key Laboratory of Pathogenic Microbiology and Immunology, Institute of Microbiology, Chinese Academy of Science, Beijing, 100101 China

The gut microbiome has attracted increasing attention over the past 15 years. Along with the fast-growing body of research literature and media reports, the public’s opinions on the gut microbiome have begun to appear polarized.

Some believe that the gut microbiome is at the core of human health and is related to every single disease. To the opposite, some people question the scientific basis of gut microbiome studies and criticize that many of them are farfetched; there is even a joke spreading in the biomedical research field—“keeping gut microbiome in mind, no mechanism is hard to find”.

However, neither view is impartial. Here, we present seven facts about the gut microbiome research, and provide five initiatives to promote the healthy development of this field.

## FACT 1. THE GUT MICROBIOME IS THE LEADING EDGE OF SCIENTIFIC RESEARCH

**Figure 1:**
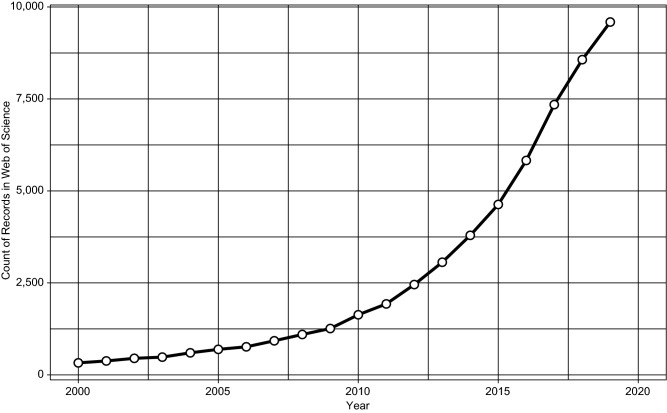
**Growth of gut microbiome studies in Web of Science database**. Query “TS = (gut OR intestine OR bowel OR intestinal OR colon OR colorectal OR gastrointestine OR gastrointestinal) AND TS = (microbiome OR microbiota OR flora OR microbe OR microbes OR commensal OR symbiont OR pathobiont OR mycobiome OR virome OR metagenome OR meta-genome)” and then retrieve those published from 2000–2019. Database accessed on Dec. 18, 2019

There are more than 50,000 gut microbiome research articles in the database of Web of Science since 2000. Prior to 2005, the relevant publications averaged no more than 500 per year. However, as of December 18, 2019, there are already more than 9,500 publications year-to-date, which has grown about 30 times since 2000, 6 times since 2010, and doubled since 2015 (Fig. 1).

Among these publications, more than 2,000 are highly cited (the top one percent in each of the 22 ESI subject areas per year), published in 449 journals. The top 15 journals with the most highly cited articles are *Nature*, *Gut*, *Science*, *PNAS*, *Cell Host* & *Microbe*, *Gastroenterology*, *Cell*, *PloS One*, *ISME Journal*, *Nature Communications*, *Nature Reviews Microbiology*, *Nature Reviews Gastroenterology* & *Hepatology*, *Nutrients*, *Immunity*, and *Nature Medicine*.

**Figure 2:**
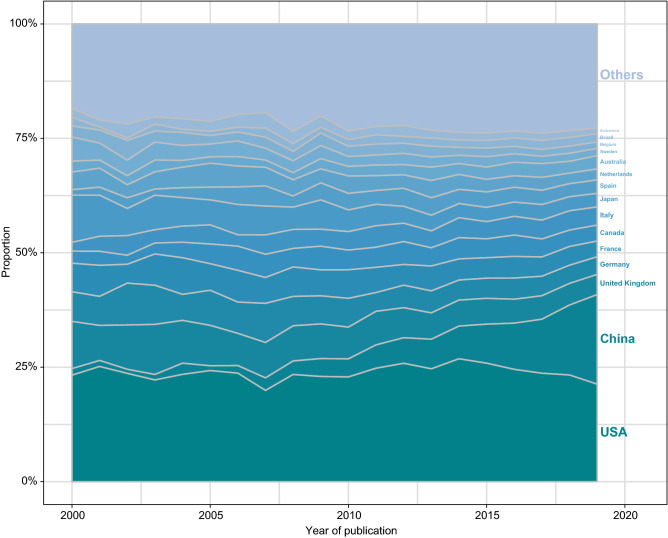
**Proportion of country contributions**

More than 170 countries and regions around the world contributed to these highly cited studies, with universal participation by major countries including the USA, China, United Kingdom, Germany, France, Canada, Italy, Japan, Spain, Netherlands, Australia, etc. Among them, China’s contribution (including Hong Kong, Macao and Taiwan) has made considerable progress in the past decade (Fig. 2).

Behind the rise of gut microbiome research is the rapid development of cutting-edge biological research technologies (Gilbert et al., 2016; Gilbert et al., 2018), including germ-free animal models (Uzbay, 2019), next-generation sequencing technologies (Shendure et al., 2017), and multi-omics approaches (Quince et al., 2017; Lagier et al., 2018; Zhang et al., 2019) such as metagenomics, metatranscriptomics, metabolomics, and culturomics. The development and application of these technologies not only enable researchers to analyze composition and structure of gut microbiome, but also make it possible to study and verify the function of the microbiome and its association with health and disease from different interdisciplinary perspectives.

## FACT 2. BASIC AND TRANSLATIONAL RESEARCH OF THE GUT MICROBIOME IS EXPANDING GLOBALLY

The rapid development of the gut microbiome research in recent years is not only related to the scientific community’s deepening understanding of the function of the microbiome, but also inseparable from each nation’s strategic support.

Of these, the Human Microbiome Project (HMP) supported by the United States’ National Institutes of Health (NIH) is best known. Between 2007 and 2016, NIH invested a total of $ 215 million in HMP (Proctor, 2019), with an additional $ 728 million for other human microbiome studies from 2012 to 2016 (NIH Human Microbiome Portfolio Analysis Team, 2019). China, the United Kingdom, Germany, Canada, France, and other countries and regions also continue to increase investment. It is estimated that the global investment in microbiome-related research over the past decade has exceeded $ 1.7 billion (Proctor, 2019).

**Figure 3:**
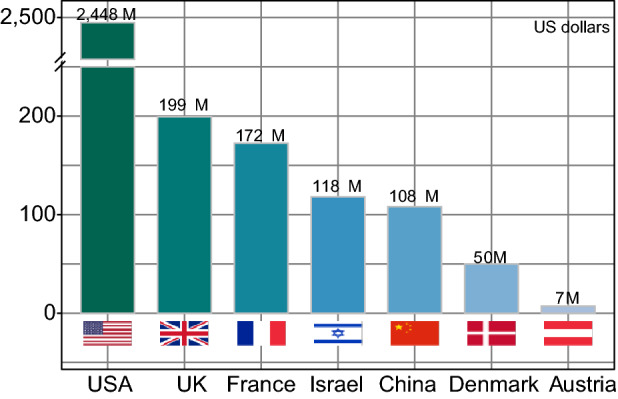
**Financing of gut microbiota-related companies in different countries (from public data)**

At the same time, industrialization and commercialization of gut microbiome-related application is also progressing. The establishment of related biotechnology companies has sprung up, with considerable global investment. As of 2019, more than $ 3 billion has been invested in gut microbiome-related innovation companies. Similar to basic research, the United States took the lead with more than $ 2.4 billion in investment, and other countries are catching up (Fig. 3).

## FACT 3. THE GUT MICROBIOME CONTRIBUTES TO HEALTH AND DISEASES

The pivotal role of the gastrointestinal (GI) tract in health forms the basis for the gut microbiome to participate in host physiological processes and even affect human health and disease (Goldszmid and Trinchieri, 2012). Besides its role in the digestion, absorption, and metabolism of food and nutrients, the gut is an important immune and endocrine organ (Rehfeld, 1998; Weiner et al., 2011; Gribble and Reimann, 2019). In addition, it is also known as “the second brain” with a complex enteric nervous system which communicates with the brain via the vagus nerve (Mayer, 2011; Avetisyan et al., 2015; Yoo and Mazmanian, 2017).

Gut microbiome studies are now transforming from correlation to causation, with the efforts to address the mechanisms by which the microbiome influences host health (Gilbert et al., 2018; Schmidt et al., 2018). In their *Nature* paper published in 2006, by using fecal microbiota transplantation in mice, the Gordon lab demonstrated for the first time that host obesity phenotype can be affected and transmitted by the gut microbiome (Turnbaugh et al., 2006).

Since then, growing evidence confirms that the gut microbiome can be considered the “microbial organ” of the body (O’Hara and Shanahan, 2006; Byndloss and Bäumler, 2018), acting through multiple mechanisms (e.g., microbiota-derived metabolites (Schroeder and Bäckhed, 2016; Dalile et al., 2019) and microbial translocation (Manfredo Vieira et al., 2018; Meisel et al., 2018)) to communicate with the host and regulate host physiology at both local and systemic levels. It is believed that the gut microbiome can affect host metabolism (Tilg et al., 2020), immunity (Belkaid and Harrison, 2017), endocrine (Rooks and Garrett, 2016; Rastelli et al., 2019), and neural function (Sharon et al., 2016) and thus contribute to host susceptibility to a series of diseases (Sekirov et al., 2010; Clemente et al., 2012; Nicholson et al., 2012; Lynch and Pedersen 2016), such as obesity (Le Chatelier et al., 2013; Ridaura et al., 2013), diabetes (Qin et al., 2012), fatty liver diseases (Canfora et al., 2019), cardiovascular diseases (Schiattarella et al., 2017), autoimmune and inflammatory diseases (Clemente et al., 2018), psychological and neurological disorders (Cryan et al., 2020), and cancer (Garrett, 2015; Yu and Schwabe, 2017; Vitiello et al., 2019).

In addition to the discovery of pathology and disease mechanisms, gut microbiome research also promotes the development of novel diagnosis and therapeutic interventions (Gilbert et al., 2018).

Gut microbiota-related biomarkers can potentially be used for colorectal cancer and adenoma screening, and can increase the accuracy of current non-invasive screening methods when used combinedly (Wong and Yu, 2019). Restoration of the gut microbiome by fecal microbiota transplantation (FMT) has proven effective in treating recurrent *C*. *difficile* infection and is also a potential treatment for inflammatory bowel disease and other diseases (Vaughn et al., 2019). Dietary fiber intervention can improve type 2 diabetes through selectively promoting short-chain fatty acid-producing bacteria (Zhao et al., 2018); and microbiota-directed complementary food seems a promising approach for treating childhood undernutrition (Gehrig et al., 2019).

The gut microbiome is also a key factor in personalized medicine. For example, a machine-learning algorithm that integrates gut microbiota and other anthropometric parameters can predict personalized postprandial glycemic response (Zeevi et al., 2015). In addition, targeting specific overgrowing pathobiont is a promising strategy to treat the corresponding disease (Duan et al., 2019; Yuan et al., 2019).

Furthermore, gut microbiome can also influence the effect of drugs and therapies by participating in drug metabolism and affecting host immune response (Skelly et al., 2019; Zimmermann et al., 2019a, b); and it has been shown that lab animals that are too “clean” are not optimal models for drug development (Rosshart et al., 2019).

## FACT 4. GUT DYSBIOSIS IS ONLY ONE FACTOR IN DISEASE

Although plenty of work has shown that the gut microbiome plays a crucial role in human health, other factors, such as genetics, environmental exposure, and lifestyle, can also influence disease risk (Franks and McCarthy, 2016; Hall et al., 2017; Gentile and Weir, 2018; Scott et al., 2019). It has been speculated that, in some cases, gut microbiome abnormality may be not the root cause of disease but rather a mediating factor or a bystander (Hanage, 2014; Franks and McCarthy, 2016; Crow, 2018; Inamo, 2019; Jia et al., 2019).

For diabetes, obesity, and other metabolic diseases that are closely related to the gut microbiome (Qin et al., 2012; Le Chatelier et al., 2013; Ridaura et al., 2013; Komaroff, 2017; Schiattarella et al., 2017), it is still unclear how much the microbiome contributes to disease initiation and progression. The development of these diseases is more likely a combined result of different driving factors (Keith et al., 2006; Franks and McCarthy, 2016; Chatterjee et al., 2017), including genetic susceptibility, dietary habits, physical activity, early life influences, sleep, and medication.

Food allergy is another condition that correlates with changes in the gut microbiome (Iweala and Nagler, 2019). There are reports that microbiome-based interventions, such as probiotics, prebiotics, and fecal microbiota transplantation, may help reduce symptoms (Bunyavanich, 2019). However, the risk of allergic disease is also influenced by a series of factors (Iweala and Nagler, 2019). In addition to genetic predisposition, other early life factors including caesarean section, preterm birth, antibiotic usage, environmental pollution, infections, and maternal smoking and excessive drinking can all contribute to the development of food allergy (Reynolds and Finlay, 2017; Mitselou et al., 2018; Levin et al., 2019) and need to be considered for disease prevention and treatment (Tordesillas et al., 2017).

When talking about gut microbiome-associated diseases, such as diarrhea (Singh et al., 2018), inflammatory bowel disease (Piovani et al., 2019), irritable bowel syndrome (Ford et al., 2017), cardiovascular disease (Miller et al., 2017), autism (Bai et al., 2019), Alzheimer’s disease (Kivipelto et al., 2018), Parkinson’s disease (Ascherio and Schwarzschild, 2016), it is important to keep in mind that the gut microbiome is only one aspect of the disease, and we should avoid overemphasizing its importance.

## FACT 5: GUT MICROBIOME RESEARCH IS STILL AT ITS INFANCY

What we know so far about gut microbiome might still just be the tip of the iceberg, and many limitations, inconclusive findings and controversies still exist within this field.

Inter-personal gut microbiome is highly individualized, and even within the same individual, gut microbiome is highly dynamic within short term, thus we still lack a good definition of “healthy microbiome”. This missing baseline data or definition also hinders many of the translational and interventional research (Lloyd-Price et al., 2016).

Besides bacteria, we also need to consider the impact of phages (Shkoporov and Hill, 2019), archaea (Pennisi 2017) and fungi (Richard and Sokol, 2019) in the GI tract on host health and diseases. Researches into those topics are still at a relative early stage. How do we study their function and potential roles of vast unknown and/or uncultured microbes? Metagenomics and culturomics are improving constantly and facilitate such analyses, yet many remain to be done (Nayfach et al., 2019).

Also, can animal studies be translated directly to humans? Probably not, as recently the US firm Synlogic has created an engineered bacteria strain and treated phenylketonuria in mouse models (Isabella et al., 2018), yet it failed in the human clinical trial, suggesting a direct and simple translation is not feasible.

How to resolve the standardization and repeatability issues in microbiome research? Such essential issues call for even more scientists to work at a global scale for standardization in methodology and more open databases to be shared among researchers (Servick, 2016; Costea et al., 2017; Poussin et al., 2018; Schloss, 2018).

How about long-term safety in microbiome intervention approaches such as prebiotics, probiotics and fecal-material transplantation? Recent researches have indicated that many of the interventions carry potential risks (Singh et al., 2018; Suez et al., 2018; DeFilipp et al., 2019; Yelin et al., 2019). And how do we manipulate microbiome with precision? One example might be personalized nutrition research and translation (Zmora et al., 2016; Johnson et al., 2019) and it has gained attention, yet this has to be done on large number of individuals and to be tracked over a longer time.

Thus, it is fair to conclude that microbiome research is still in its infancy, more thorough research with better methods has to be carried out to determine the role as well as mechanisms of gut microbiome in human health and diseases, plus the efficacy and safety of interventional approaches.

## FACT 6: CHAOS AND CONFLICT OF INTEREST EXIST IN GUT MICROBIOME RESEARCH

Due to inappropriate self-publicity and interpretation of certain researchers, combined with the further exaggeration of many media and commercial partners, many of the gut microbiome research results and microbiome-based research products have been labeled erroneously to be the new panacea, capable of treating all kinds of illness. This idea was even adopted by many of the radical researchers. Such extreme kind of concept is for sure detrimental for the gut microbiome research and industry.

Currently, the golden standard for evaluating drug safety and efficacy is randomized, controlled clinical trials (Bothwell and Podolsky, 2016; Djulbegovic and Guyatt, 2017), which has also been used to evaluate gut microbiome related interventions. Yet in reality, randomized controlled trials (RCTs) are limited by individual differences, short term of intervention etc when it comes to evaluating prebiotics and probiotics (Zeilstra et al., 2018), and functional evidence is also limited to deepen the insights into mechanisms (Guarino and Canani, 2016).

In such context, exaggeration of the probiotics’ efficacy, misplacing the function of whole gut microbiome by just a few products, and abuse of research products, as well as ignoring the complexity of gut microbiome by over-simplifying study design and interventional approaches are among the common phenomena in this field (Crow, 2018).

In addition, in commercial activities there are cases of promoting probiotics towards immune-deficient populations, which is potentially risky (Cohen, 2018). In fact, special population including ICU patients (Yelin et al., 2019) and hospitalized elderly (Dauby, 2017) are at risk of bacteraemia when intake probiotics, and in extreme cases, immune-deficient elderly could develop hepatapostema and bacteraemia after long-term ingestion of yogurts (Pararajasingam and Uwagwu, 2017).

Also, when the essential question remains unanswered regarding the baseline healthy microbiome’s characteristics, the concept of gut microbiome dysregulation or dysbiosis has been abused, to lure the consumers and patients to purchase microbiome-related products (Shanahan and Hill, 2019).

Besides, we need to be alerted by the conflict of commercial interests within research. For instance, food industry sponsored nutritional research (Mozaffarian, 2017), or even those by public health research organization (Galea and Saitz, 2017), professional associations or societies (Nissen, 2017), those who set the standards (Sox, 2017) all could be affected by different interests (Greenhalgh, 2019). Nutritional analyses and meta-analyses might also be manipulated to cover the negative effects of certain products (Barnard et al., 2017).

Under such circumstances, many of the claims by infant dairy products including promoting cognitive capacity and safeguarding intestinal health lack sufficient scientific evidence (Hughes et al., 2017). Yet how to regulate such issues remains a difficult task for regulatory bodies including the US FDA (The Lancet null, 2019).

## FACT 7: SELF-PURIFICATION AND DISCIPLINE MECHANISMS EXIST IN GUT MICROBIOME RESEARCH

Many of experts in this field have realized that the recent zealousness might lead to a topic bubble, and the call for strengthening self-purification and discipline also gained support, with a few such recommendations being issued via scientific journals.

First of all, experts call for the principle of caution and critics regarding microbiome research (Hanage, 2014; Crow, 2018). For instance, the description of research results has to be accurate and faithful, and avoid over-interpretation regarding non-causal studies as well as animal studies. While accumulating evidence over causality, we also need to deepen the microbiome research into the level of bacterial strains and bacterial genes, to gain insights onto the exact mechanisms (Fischbach, 2018). This is particularly important for the field of probiotics (Kleerebezem et al., 2019). Plus, researchers are also working on the standardization of procedure and methodology in microbiome research (Knight et al., 2018).

Another new task is to regulate effectively all the diverse upcoming approaches in microbiome intervention (Aagaard and Hohmann, 2019). Many recommendations were issued regarding interventional approaches including probiotics with potential risks (Cohen, 2018) and FMT (Hoffmann et al., 2017; Verbeke et al., 2017), as well as novel food products targeting microbiome and aiming at microbiome health (Green et al., 2017).

The public are expected to understand, appreciate and eventually participate into the microbiome research and translation, while the research and translation themselves have to effectively avoid risk and safety issues (Dominguez-Bello et al., 2016; Shamarina et al., 2017). It is widely accepted that ethics has to be obeyed during the process of research and translation (O’Doherty et al., 2016; Rhodes 2016).

It is not without solution when it comes to the complex, multi-faceted conflict of interests issues, as pointed out by scholars (Stead, 2017). For instance, many of the recommendations have been made that the health policy making process should not be influenced by food industries’ interests (Untangle food industry influences on health 2019), scientific researchers should disclose actively and fully their own potential conflict of interest (Thornton, 2017), and medical professionals should disclose such conflicts to patients as well (Zuger, 2017).

Thus, it is obvious that a fully functioning self-purification and discipline mechanism has been established among the scientific bodies of microbiome researchers, covering the design and execution of research, means of regulation and biological ethics.

## FIVE INITIATIVES

Based on the above 7 facts about gut microbiome, in particular, we propose below five initiatives for basic researchers, clinicians, and industrial professionals in relevant fields:

### Follow the normative ethical principles to carry out research

We propose that research activities related to gut microbiome should be subject to necessary ethical review and peer review.

Researchers should learn, practice and respect bioethical principles, adhere to scientific spirit, scientific culture and research ethics; on the basis of truth-seeking and rational criticism, researchers should strive to be innovative and responsible; researchers should adjust the relationship between themselves and collaborators (including other researchers, funders, research subjects, general public/consumers) and objects (including experimental animals, ecological environment, etc.) in a timely manner, and also take social responsibility.

### Avoid hype and packaging

We propose that basic researchers, clinicians, industry and media professionals should avoid hype and over-promoting/packaging of gut microbiome and themselves, and actively participate in self-discipline and self-purifying in the whole field.

Researchers should avoid using their affiliated insititutions, and their position endorsement to create personal influence, refrain from self-marketing, and also avoid exaggerating or over-promoting individual or team’s research results. In the publicity work of scientific research, industry, and social media, we should avoid the labeling gut microbiome as “panacea” or “useless”; we oppose opportunism, clickbait, and sensationalising the hot topic; we oppose the “vulgarization” and “pseudoscientization” of gut microbiome research, including the abuse of the concept of “gut microbiome” to irrelevant fields, or promise unrealistic effects, etc.

### Disclose conflict of interest and reveal safety and risk issues actively

We propose that researchers actively disclose the conflict of interest in various occasions, such as publishing papers, academic speeches, media publicity, etc., and at the same time, if there are potential safety risks in related products and services, they should proactively inform consumers or patients.

In both research and translational application, it is important to maintain openness and transparency during risk recognition, assessment, risk management and damage control. Researchers actively disclose conflict of interest would not only be beneficial for individuals, but also for the brand accumulated value and dissemination of products and/or services. Meanwhile, if researchers can actively inform patients/ consumers about the safety risks, this will also increase brand trusts and credibility of individuals, products or services, and effectively avoid bringing risks to consumers/patients, thus reducing the systematic risks of whole industry.

### Abide by laws and disciplines and adhere to evidence-based scientific rules

We propose to strictly abide by national laws and regulations and adhere to evidence-based scientific rules in the process of industrialization. The function, efficacy, and health benefit claims of a specific product or service must comply with the relevant national laws and regulations, and must never break the legal and ethical bottom line. The promotion of function and efficacy of a particular products or services should be based on high-quality evidence, and the evidence of RCT should be developed and accumulated. We should not over-interpret the results generated from cell and animal experiments, whereas also pay special attention to the risks of side effects and unintended consequences.

### Actively participate in science popularization and education and promote public participation

We call on professionals to actively participate in science popularization, dissemination and education activities for the public, meanwhile also consciously absorb public opinions and encourage the public to participate in and contribute to scientific research equally.

In the face of the rapid development of gut microbiome research and fast growing of knowledge update and accumulation, there are a mix of hope, help, and hype, which sometimes make people confused. Furthermore, the area of gut microbiome is increasingly influenced and even dominated by commercial market and capitals, which may put public interest at stake. Therefore, established and mainstream front-line experts are urgently needed to participate in popular science activities. In the daily science communication work, professionals can systematically popularize the relevant knowledge of gut microbiota, whereas in case of big events, they should respond to the public’s concerns in a timely and transparent manner and ultimately to achieve a responsible and sustainable supportive environment for research.
